# Oral Presentations - Abstracts of the 26^th^ Annual Congress of the SBRA. São Paulo/SP - Brazil, 2022

**DOI:** 10.5935/1518-0557.20220045

**Published:** 2022

## O-01. Artificial Intelligence applied for ploidy prediction through blastocyst’s morphological assessment

**Catherine Kuhn Jacobs^1^, Mariana Nicolielo^1^, Dóris Spinosa Chéles^1^, Paula Luiza Mendes de Chico^1^, Bruno Araújo Mendes^2^, Matheus Bubola Duarte^2^, Mauricio Barbour Chehin^3^, José Roberto Alegretti^1^, Eduardo Leme Alves da Motta^3,4^, Marcelo Fabio Gouveia Nogueira^2^, José Celso Rocha^2^, Aline Rodrigues Lorenzon^5^**

*^1^Department of Embryology - Huntington Medicina Reprodutiva - Eugin Group, São Paulo, SP, Brazil*.

*^2^Department of Biological Sciences - School of Sciences and Languages, São Paulo State University, Assis, SP, Brazil*.

*^3^Clinical Department - Huntington Medicina Reprodutiva - Eugin Group, São Paulo, SP, Brazil*.

*^4^Department of Gynecology - Paulista School of Medicine, Federal University of São Paulo, São Paulo, SP, Brazil*.

*^5^Scientific Coordinator - Huntington Medicina Reprodutiva - Eugin Group, São Paulo, SP, Brazil*.

**Objective:** Embryo quality and implantation potential have consistently been evaluated and predicted by morphology. Even with the implement of more complexes approaches as preimplantation genetic testing for aneuploidy (PGT-A) and most recently, the time- lapse incubators and morphokinetics evaluation, morphology is still prioritized in embryo selection. The development of artificial intelligence (AI) algorithms able to extract features from an image of the embryo and correlate to crucial information, as ploidy, is seen as a goal for many researchers in the field. Considering that embryo aneuploidy is known as the main cause of embryo implantation failure and miscarriage and its most reliable diagnose is costly and invasive, this study aims to verify an AI algorithm performance to distinguish between euploid to aneuploid blastocysts using single 2-D embryo images.

**Methods:** This is a prospective cohort study with data collected from 858 biopsied embryos from IVF cycles of 325 patients undergoing PGT-A (NGS platform) after informed consent form signature between June 2019 and March 2022. Embryos were cultured in a time-lapse system incubator (EmbryoscopePlus, Vitrolife) until blastocyst stage, biopsied and followed vitrification or transfer as medical referred. A single 2-D image of the expanded blastocyst with the inner cell mass and trophectoderm well visualized was obtained from the time-lapse incubator software and anonymously sent to be analyzed. In the laboratory of applied and computational mathematics the images were digitally processed and numerical parameters were automatically extracted from the morphological features of the blastocyst. These data together with the known PGT- A result (euploid or aneuploid) were randomly divided and used for the training (70%), validation (15%) and testing (15%) of an AI algorithm developed by association of artificial neural networks (ANN) and genetic algorithms (GA) for ploidy assessment. Later on, for the blind-test, the database was checked only after AI algorithm was tested. The area under the curve (AUC) of the receiver operating characteristic (ROC) curve was measured to obtain predictive power.

**Results:** Among the 858 biopsied embryos, 421 were euploid and 437 were aneuploid embryos. The AI algorithm was trained with 535 embryos (AUC for euploid=0.98 and aneuploid= 0.99), tested with 115 embryos (AUC for euploid=0.70 and aneuploid=0.71) and validated with 115 embryos (AUC for euploid=0.70 and aneuploid=0.69), computing 765 embryos. For the blind-test 93 embryos were used (AUC for euploid=0.72 and aneuploid=0.74).

**Conclusion:** The AI algorithm could estimate the embryo status in euploid or aneuploid using an image analysis with an accuracy of 0.98 and 0.99 in the training and 0.72 and 0.74 in the blind-test, respectively. This result brings the perspective of a non-invasive and highly effective approach addressing embryo ploidy to be considered while selecting the best embryo to transfer.

## O-02. Normal embryo development and early pregnancy resulting from ICSI of vitrified-warmed *ex vivo* retrieved mature oocytes in a woman with bilateral ovarian carcinoma

**Bruno Ramalho de Carvalho^1^, Íris de Oliveira Cabral^2^, Taise Moura Franceschi^1^, Geórgia Fontes Cintra^3^, Leandro Santos de Araújo Resende^4^, Janina Ferreira Loureiro Huguenin^5^, Luiz Eduardo de Almeida Prado Franceschi^6^, Andrea Tatiane Oliveira da Silva Barros^7^**

*^1^Bruno Ramalho Reprodução Humana, Brasília, DF*

*^2^GENESIS Centro de Assistência em Reprodução Humana, Brasília, DF*

3 Hcor Oncologia, São Paulo, SP

*^4^Oncoclínicas Aliança Asa Sul, Brasília, DF*

*^5^Instituto de Câncer de Brasília, Brasília, DF*

*^6^Diagnose, Laboratório de Anatomia Patológica e Citologia, Brasília, DF*

*^7^Centro de Tratamento do Câncer Medradius, Maceió, AL*

**Objective:** To report normal embryo development and early surrogate pregnancy resulting from intracytoplasmic sperm injection (ICSI) after ex vivo retrieval of in vivo matured oocytes, in a woman with bilateral ovarian carcinoma, submitted to total abdominal hysterectomy and bilateral salpingo- oophorectomy.

**Methods:** Case report, ex vivo retrieval of mature oocytes after ovarian stimulation.

**Results:** A 34-year-old nuligesta presented with bilateral ovarian tumor, referred by the oncologist for planning fertility preservation. According to the International Ovarian Tumor Analysis (IOTA) Assessment of Different NEoplasias in the adneXa (ADNEX) model, the risk of malignancy at 96.1%. Follicular aspiration by the conventional transvaginal technique was considered unsafe, due to the risk of tumor cell spillage. Therefore, controlled ovarian stimulation (COS) was proceeded from the second day of menstrual cycle, by the administration of corifollitropin alfa 150 mcg (Elonva, Schering-Plough); daily ganirelix acetate 0.25 mg was added from COS Day 9 to Day 12; daily recombinant follitropin 200IU (Puregon, Schering-Plough) was administered from Day 8 onwards, to complete COS, and final maturation was achieved by a single dose of choriogonadotropin alfa 25 mcg, on Day 12. Follicular puncture occurred in the operating room, immediately after oophorectomy (maximum ischemia time of 31 minutes), 36 hours after the trigger, guided by ultrasound, with a linear transducer applied directly to the specimens. Twenty-one ovarian follicles were aspirated, and 13 oocytes were collected, 12 of which were mature (MII) and then, vitrified by the Cryotec method (Reprolife, Japan). Seventeen months later, after completing neoadjuvant cancer treatment and with no recurrency to date, the patient returned, desiring to become a mother, and counting on her 33-year- old sister as a surrogate mother. ICSI with anonymous donor sperm was performed in the 12 MII warmed oocytes, resulting in the normal development of 9 embryos (Table 1) cultured in time-lapse incubators (EmbryoScope+, Vitrolife, Sweden), using single step medium (G-TL, Vitrolife, Sweden), two of which were chosen to be transferred to the surrogate uterus, according to the morphokinetic- based KIDScore Day 3 (Vitrolife, Sweden) for prediction of implantation. Surplus embryos were cryopreserved. A positive beta-human chorionic gonadotropin was detected twelve days after transfer (149 mIU/mL) and the expected elevation of its level was confirmed after ten days (15.481 mIU/mL).

**Conclusion:** Vitrification of oocytes should be the strategy of choice for preserving female fertility, but in cases of ovarian cancer, transvaginal follicular aspiration increases the risk of malignant cells spillage into the pelvis. To offer a safe alternative for those cases, aspiration of stimulated ovaries immediately after oophorectomy eliminates the risk of altering the prognosis of the disease. In this report, we point out the efficiency of the technique and the viability of mature oocytes retrieved ex vivo, whose ICSI, after vitrification and warming, resulted in normal embryo development and early pregnancy.

**Table 1:** Cleavage-stage (Day 3) embryos’ development and classification.

Oocyte^1^	Embryo Description	Morphological Grade^2^	Decision
1	Score 2	2PN; 7cG1	Freeze
2	Score 5	2PN; 8cG2	Freeze
3	Score 5	2PN; 9cG1	Transfer
5	Score 1	2PN; 6cG3	Freeze
6	Score 2	2PN; 9cG1	Freeze
7	Score 5	2PN; compacting, G2	Transfer
9	Score 2	2PN; 8cG1	Freeze
10	Score 2	2PN; 8cG2	Freeze
11	Score 5	2PN; 8cG2	Freeze

PN = pronuclei; c = cells; G = grade.

## O-03. Birth weight and prematurity rates according to maternal age in 3724 *in vitro* fertilization (IVF) cycles

**Aline R. Lorenzon^1^, Jorgelina C. Razeto^2^, Catherine Jacobs^1^, Mariana Nicolielo^1^, Claudia Gomes^1^, Thais Sanches Domingues^1^, Mauricio Barbour Chehin^1^, José Roberto Alegretti^1^**

*^1^Huntington Medicina Reprodutiva, Eugin Group;*

*^2^Universitat Pompeu Fabra, Barcelona, Espanha*

**Objectives:** The use of ART (assisted reproductive technologies) is increasing yearly worldwide and this is mostly due to the postponement of motherhood because the new role of women in society and their new priorities in relation to family planning. The use of ART may lead to pregnancy achievement; however, it is also important to understand the perinatal outcomes of these advanced maternal age women. The objective of this retrospective cohort study was to investigate if the birth weight and prematurity of singleton newborn from ART treatments were correlated to maternal age.

**Methods:** We included patients that underwent *in vitro* fertilization cycles at Huntington Reproductive Medicine Center in Sao Paulo, Brazil and had a singleton live birth (>22 weeks) with fresh or frozen embryo transfer (a total of 3724 patients). Both autologous and donor cycles were considered. The period analyzed covered January, 1^st^ 2012 to December, 31^st^ 2018. Baseline maternal and neonatal characteristics included maternal age at cycle start, origin of oocyte (autologous or donor), IVF or ICSI as fertilization technique, fresh or frozen embryo transfer, embryo stage (cleavage or blastocyst), PGT-A (yes or no), birth weight (BW, grams), gestational age at birth, sex of live birth and maternal body mass index (BMI) at cycle start. The main studied outcome was BW (adjusted for GA at delivery), and the secondary outcome was prematurity (<37 weeks). Marginal statistical analyses were performed accordingly (t test, Mann-Whitney, Kruskal-Wallis, chi-square test) and a linear regression model was carried out to calculate the effect of maternal age on birth weight and prematurity. The significance level adopted was *p*<0.05. Data analysis was performed using R software, version 4.2.0

**Results:** From the studied population (3724 women), 2899 patients (77.6%) underwent autologous cycles and 836 (22.4%) received a donor oocyte. The distribution of maternal age at cycle start according to the Society of Assisted Reproduction Technology (SART) classification was: <35 years (32.40%), 35-37 (26%), 38-40 (20.6%), 41-42 (6.75%) and > 42 years (12.6%). Mean maternal age in autologous cycles was 35.29±3.80 years and 42.07±4.65 in donor cycles. Maternal BMI were similar in autologous and donor cycles (23.7±3.5 versus 23.9±3.2). The majority of newborns were from embryos conceived by ICSI (89.5%), transferred in blastocyst stage (90.9%) in frozen cycles (60.6%), without biopsy for PGT (81.6%) and were male (51.9%). Regarding gestational age, age groups <35, 35-37 and 38-40 are statistically equal (mean of 38.4, 38.4 and 38.2 weeks respectively). The age group 41-42 (mean 38.0 weeks) is statistically different from the age groups <35, 35-37 and statistically equal to 38-40 (p=0.0946). The age group >42 (mean 37.57 weeks) is statistically different from the others and has the lowest mean gestational age at delivery (p=0.005). In the BW analysis, age groups <35, 35-37 and 38-40 are statistically equal (BW mean 3127.57, 3100.35, 3081.0 grams respectively). Age groups 41-42 and >42 (3031.22, 2994.65 grams respectively) are statistically different from the age group <35 (*p*=0.02 and 0.002 respectively) and statistically similar to the others. Patients older than 42 years old had an overall of 20.5% prematurity rate, in contrast to 13.3% in patients younger than 35 and between 35-37, 15.0% and 16.6% in patients between 38-40 and 41-42 (*p*≤0.0001). After adjustment for confounders, the regression model showed that patients older than 42 years had lower BW babies and higher incidence of prematurity, regardless of the origin of oocyte (autologous or donor).

**Conclusions:** It is important to highlight that although ART may surpass infertility in advanced maternal age women, obstetrical and perinatal outcomes may reflect on future health of offspring. In this way proper advice should be included in prenatal care of these patients.

## O-04. Effect of propranolol on primordial follicle activation in mice

**Stella Pollyanne de Oliveira^1^, Bernardo Camara do Nascimento^1^, Paulo Henrique de Almeida Campus Junior^1^**

*^1^Universidade Federal de São João del Rei*

**Objective**: The aim of this study was to evaluate the effect of in vivo treatment with propranolol on ovarian follicular activation and mobilization until ovulation, on follicular dynamics and body weight in female C57BL6 mice.

**Methods:** Animals of the C57BL6 strain at 6 weeks were divided into three groups: control, low and high (n=6 per group). The animals in the control group received the vehicle (PBS), those in the Low group received a dosage of 15 mg/kg and those in the High group received a dosage of 40 mg/kg, all applied intraperitoneally for 15 consecutive days. The body weights of the females were measured every day at the time of application of the treatment. The estrous cycle analysis was done through vaginal cytology, the estrous cycle stage was determined based on the presence or absence of leukocytes, cornified epithelial cells and nucleated epithelial cells. At the end of the experimental period, the ovaries were collected and destined for histological preparation and follicular quantification. With these analyses, the number of primordial, primary, secondary, antral and atretic follicles, as well as the rates of activation and follicular atresia, were obtained. For the analysis of superovulation, the animals were submitted to the same procedures and were administered with 20 IU of eCG, after 48 hours 20 IU of hCG and after 14 hours the ovulated oocytes were collected and quantified (n=10 for each group). Data were submitted to analysis of variance, followed by the Newman-Keuls test, with the aid of the GraphPad Prisma 8.

**Results:** The duration of the estrous cycle was not altered by the treatments (p>0.05) and the body weight was also not altered by the administration of propranolol (*p*>0.05). There was a decrease in the number of primordial follicles of 23.5% in the Low group and of 35% in the High group in relation to the control (*p*=0.03) and an increase in the number of primary follicles by more than 100% in the treatments in relation to the control (*p*=0.007). In the other classes of follicles (secondary and antral) there was no significant difference between treatments in relation to the control (*p*>0.05). The treatments did not change the number of atretic follicles (*p*>0.05). The follicular activation rate showed an increase of 80% in the Low group and 95% in the High group (*p*=0.0004) in relation to the control group. However, in the rate of atresia there was no significant difference between the groups (*p*>0.05). In the superovulation assay, there were no significant differences between groups (*p*>0.05).

**Conclusion:** In general, the results indicate that Propranolol acts in the activation of primordial follicles, as it is a drug described as activating mTOR, and mTOR is possibly related to follicular activation. However, there was no change in the number of secondary and antral follicles and in ovulation, thus showing that it acts in the preantral phase. As the number of atresic follicles and the rate of atresia did not change, we believe that there was possibly a compensation for this activation. The present study brought new data to the literature regarding the activity of propranolol in the activation of primordial follicles in vivo, in addition to unprecedented results of a drug already known on the market, opening the possibility for new research and applications in the human clinic.

## O-05. Multicentre validation of CHLOE-EQ: An embryo assessment assistant based on Artificial Intelligence (AI)

**Luiz Mauro Oliveira Gomes^1^, José Fernando de Macedo^1^, Edson Borges Jr.^2^, Patricia Guilherme^2^, Isaac Moise Yadid^3^, Thelma Santos Criscuolo^3^, Ricardo Azambuja^4^, Mariangela Badalotti^4^, Bernardo Rodrigues Lamounier de Moura^5^, Fernando Prado Ferreira^5^, Alexa Zepeda^6^, Adriana Brualla^6^, Cristina Hickman^6^**

*^1^Reproferty - Centro de Reprodução Humana*

*^2^ Fertility Medical Group*

*^3^ Clínica Primordia Medicina Reprodutiva*

*^4^ Fertilitat - Centro de Medicina Reprodutiva*

*^5^ EmbrioLógica*

*^6^ Fairtility*

**Objective**: CHLOE-EQ is an embryo assessment assistant that automatically processes time-lapse videos using AI with the objective to increase consistency, efficacy of prediction whilst saving valuable embryologist time. The purpose of this study was to compare the assessment of embryoscope time-lapse videos by experienced embryologists with CHLOE-EQ (Fairtility) across four independent clinics.

**Methods**:Following culture of embryos in a time-lapse incubator (Embryoscope, Vitrolife) at four clinics (Clinic A N=147, Clinic B N=40, Clinic C N=143, Clinic D N=462); experienced embryologists prospectively assessed the number of pronucleates, morphokinetics, inner cell mass (ICM) and trophectoderm quality and determined which embryos should be utilised or discarded as per routine clinical practice. The same time-lapse videos were retrospectively assessed by CHLOE-EQ (Fairtility), blind to the human assessments. Intra-Correlation Coefficient (ICC) was used to quantify the level of agreement between Embryologist and CHLOE for morphokinetics: Very weak (0-0.2), weak (0.2-0.4), moderate (0.4-0.6), strong (0.6-0.8), very strong (0.8-1). Agreement of PN assessment by CHLOE and Embryologists was assessed using Kappa score. Efficacy of prediction of blastulation, utilisation, selection for transfer and ploidy was assessed against CHLOE-Blast Score and CHLOE EQ Score and CHLOE RANK using Binary logistic regression. Each of the assessments was analysed per clinic, and overall across all five clinics.

**Results**: As outlined in the table below, when combining the data from all clinics, all morphokinetics had a very strong agreement between CHLOE and embryologist annotations. At the individual clinic level, the lowest level of agreement was moderate for tPNa in clinic A and strong for t4 in clinic A: all other clinics had a very strong level of agreement between embryologist and CHLOE for all remaining morphokientics. The overall accuracy of PN assessment was 96%, with a kappa agreement 0.87 (very strong). Across all clinics, CHLOE BLAST Score was predictive of blastulation (AUC=0.77-0.99, *p*<0.001), whilst CHLOE EQ Score was predictive of utilisation (AUC 0.81-0.91), selection for transfer (AUC 0.70-0.85), euploidy (AUC=0.65-0.75) and CHLOE Ranking was predictive of utilisation (AUC=0.70-0.86) and selection for transfer (AUC=0.85-0.86).

**Conclusion**: CHLOE-EQ can automatically annotate morphokinetics, count pronucleates and identify blastulation with a strong level of agreement with experienced embryologists across different clinics, bringing a consistent language of embryo assessment that can be generalised to different clinics using time-lapse incubation. Incorporating AI based tools such as CHLOE in a time-lapse clinics can help improve consistency in embryo assessment, efficacy of prediction of embryo viability whilst saving valuable embryologist time.

**Table:** 

Morphokinetics	Clinic A	Clinic B	Clinic C	Clinic D	Overall
tPNa	0.503	0.973		0.809	0.808
tPNf		1.0		0.959	0.969
t2	0.889	0.932	0.997	0.927	0.917
t3	0.873	0.998	0.945	0.912	0.915
t4	0.746	0.997	0.942	0.958	0.836
t5	0.809	0.998	0.928	0.972	0.895
t6		0.999		0.950	0.958
t7		1.0		0.894	0.911
t8		1.0		0.900	0.917
t9		0.995			0.995
tM		0.954		0.903	0.912
tsB		0.998		0.981	0.983
tB	0.941	0.951	0.964	0.973	0.962
teB		0.971			0.971
PN accuracy			99% (195/ 197)	95% (606/ 641)	96% (801/ 838)

**Table:** 

CHLOE BLAST SCORE prediction of blastulation	AUC= 0.88	AUC= 0.92	AUC= 0.99	AUC= 0.77	AUC= 0.91
CHLOE EQ SCORE prediction of utilisation	AUC= 0.87	AUC= 0.81	AUC= 0.96	AUC= 0.91	AUC= 0.90
CHLOE EQ SCORE prediction of selection for transfer	AUC= 0.70		AUC= 0.85	AUC= 0.70	AUC= 0.75
CHLOE EQ SCORE Prediction of euploidy	AUC= 0.75		AUC= 0.67	AUC= 0.65	AUC= 0.65
CHLOE RANK Prediction of utilisation	AUC= 0.72	AUC= 0.70	AUC= 0.86	AUC= 0.71	AUC= 0.71
CHLOE RANK Prediction of selection for transfer	AUC= 0.86		AUC= 0.86	AUC= 0.85	AUC= 0.85

## O-06. Zymot vs. discontinuous gradient centrifugation: evaluation of fertilization rate and D3 good quality rate of sibling oocytes

**Lisiane Knob de Souza^1^, Bruna Campos Galgaro^1^, Luiza da Silva Rodrigues^1^, Gabriel Veber Moisés da Silva^1^, João Sabino Lahorgue da Cunha Filho^1^**

*^1^Insemine*

**Objective:** To compare fertilization rate and cleaving embryo good quality rate of sibling oocytes injected with Zymot or discontinuous gradient processed spermatozoa.

**Methods:** This is a pilot study with 85 sibling oocytes of IVF cycles from January to July 2022. The semen collected on the day of oocyte pick-up was divided into two samples, the first sample was processed in a Zymot device and the second sample was processed in a discontinuous gradient. Evaluation of all basic parameters as concentration, total motility, progressive and non-progressive motility were performed. Three hours after oocyte pick-up, intracytoplasmic sperm injection was carried out using spermatozoa from Zymot on half of the mature oocytes, and the other half was injected with gradient spermatozoa. Sixteen to eighteen hours after injection, fertilization was accessed through pronucleus visualization. The embryos were cultured in the same medium until day 3. On day 3, embryos were classified as A, B, C or D according to the number of cells, symmetry and fragmentation. Embryos grade A (eight cells, symmetric and a maximum of 10% of fragmentation) and grade B (7 cells, symmetric and a maximum of 10% of fragmentation) are considered good quality embryos. The statistical analysis was performed using qui- square, Mann-Whitney and T-Student tests and results were described as percentage, median [25^th^ - 75^th^ percentiles] and mean±DP respectively.

**Results:** The mean age of female patients who went under IVF cycles was 34.42±3.99 years and male age was 36.14±3.62. Forty-two oocytes were injected with spermatozoa from the Zymot device, and forty-three with spermatozoa from the gradient process, a mean of 6.1±1.46 and 6±1.91 (*p*=0.846) oocytes injected respectively. Seminal post- processing parameters were different between techniques, discontinuous gradient had a mean concentration of 100 [35-102] millions/mL whereas Zymot had a mean concentration of 4 [3-26] millions/mL (*p*=0.021), total motility 84.57%±7.43 and 99%±1.82 (*p*=0.002), progressive motility 68.85%±8.28 and 91.71±8.28 (*p*=0.005), respectively. Non-progressive motility was not significantly different between the two methodologies, with 15.71%±5.00 for gradient and 7.28%±3.27 for Zymot (*p*=0.083). Fertilization rate (74% vs 75%, *p*=0.568) and good quality rate (25% and 23%, *p*=0.958) were not significantly different between groups.

**Conclusion:** Although post-processing seminal parameters were different between the techniques favoring the usage of Zymot device, there were no beneficial effects regarding fertilization and day three good quality rates.

**Keywords:** Zymot, discontinuous gradient, fertilization rate

## O-07. Magnetic-levitation 3D culture system for ovarian tissue cultivation: a new and effective method to start a multistep culture

**Deize de Cássia Antonino Rissato^1^, Mayara Mafra Soares^2^, Paula Batista de Alvarenga^3^, Júlia Menezes Candido Silva^1^, Israel Nunes Silveira^1^, Jonathas Rodrigo dos Santos^4^, Carlos Antônio Couto Lima^5^, Luciane Carla Alberici^6^, Ana Carolina Japur de Sá Rosa e Silva^1^**

*^1^Department of Gynecology and Obstetrics, Faculty of Medicine of Ribeirão Preto, University of São Paulo, Ribeirão Preto, São Paulo, Brazil*.

*^2^Faculty of Veterinary Medicine, Federal University of Uberlândia, Uberlândia, Minas Gerais, Brazil*.

*^3^Institute of Health Sciences at Federal University of Mato Grosso, University Campus of Sinop, Mato Grosso, Brazil*.

*^4^Department of Biochemistry, Faculty of Medicine of Ribeirão Preto, University of São Paulo, Ribeirão Preto, São Paulo, Brazil*.

*^5^Faculty of Health Sciences, Universidade do Oeste Paulista - Unoeste, Presidente Prudente, São Paulo, Brazil*.

*^6^Department of Biomolecular Sciences, Faculty of Pharmaceutical Sciences of Ribeirão Preto, Ribeirão Preto, São Paulo, Brazil*.

**Objective:** The aim of this study was to evaluate the viability, mitochondrial activity, oxidation and connective tissue damage of bovine ovarian tissue cultured in three different 3D systems: magnetic levitation, alginate matrix or fibrin-alginate matrix.

**Methods:** Ovarian fragments were cultured *in vitro* with nanoparticle of gold and iron oxide that was cross-linked by poly- L-lysine (magnetic-levitation) or alginate 1% or fibrin-alginate 1%. The fragments were subdivided in fresh-control (D0), day 1 (D1) and day 7 (D7) after culture. The fragments were evaluated for signs of cell degeneration (propidium iodide method), mitochondrial activity (MitoTracker Orange CMTMRos technique) and intercellular levels of production of reactive oxygen species (ROS) (DCF method). All markers were analyzed by confocal microscopy and measured as the numbers of pixels obtained, controlling for background staining. After averaging the pixels of the images obtained by the confocal microscope, each fragment was analyzed at 5 different points and at each point, the 3 probes (propidium iodide, MitoTracker Orange CMTMRos and DCF) were evaluated. The quantification of oxygen consumption was performed using high resolution respirometry (Oroboros Instruments-oxygraph-2k) daily during the 7 days of culture. The respirometry analysis included: routine oxygen consumption state, the leak of oxygen consumption state and the electron transport chain (ETS) oxygen consumption state. Ovary tissues were fixed in 10% buffered formalin, routinely dehydrated and embedded, sectioned at a 5 µm thickness, and stained with Sirius Red for 1 h. After washing with water for 10 min, sections were dehydrated and sealed. Images for analysis were obtained by polarization microscopy and overall collagen contents were analyzed with ImageJ software. Two analyzes were performed for each variable: 1) One-way ANOVA for comparisons in relation to the Control group; 2) Two-way ANOVA to assess the effects of treatments (3D culture by magnetic levitation, alginate and alginate with fibrin) and days (1 and 7).

**Results:** *Tissue viability:* magnetic-levitation system presented similar degeneration than the fresh control after 7 days of culture (*p*>0.05). Fibrin-alginate presented increased degeneration in D7 (*p*<0.05) and alginate remained similar to fresh-control in D1 and D7 (*p*>0.05). *Mitochondrial activity:* the magnetic-levitation system had similar results (*p*>0.05) to the fresh-control in D1 and D7, while fibrin-alginate had lower mitochondrial activity in relation to fresh-control and to the magnetic-levitation group in D7 (*p*<0.05). *Production of ROS:* all groups including fresh- control presented similar ROS production (*p*>0.05). *Respirometry:* Magnetic-levitation presented higher routine values when compared to alginate in D1, D2 and D3. When we evaluated ETS, we observed that magnetic-levitation maintained the same parameters during the 7 days of culture, showing a decrease only in D4. The alginate and fibrin-alginate groups showed a decrease in ETS in D3 and D4, respectively, compared to D1. In general, all groups showed a decrease in routine and leak from D2 onwards compared to D1. *Sirius red staining:* we observed that the D1 and D7 of the magnetic levitation group were similar (*p*>0.05) to the fresh control and there was no difference (*p*>0.05) between the days (D1 and D7). When evaluating the fibrin-alginate matrix group A, we found that D1 presented more damage (*p*<0.05) to the connective tissue when compared to D7. However, D7 was similar to D0 (p>0.05). D1 had greater damage (*p*<0.05) of connective tissue when compared to D7. The Alginate matrix showed greater damage (*p*<0.05) in D1 and D7 when compared to the fresh control. Days D1 and D7 were similar when compared.

**Conclusion:** Ovarian tissue cultured in a magnetic levitation system showed better viability and mitochondrial activity, lower ROS production and connective tissue damage compared to control and other 3D systems.

